# Diagnostic variation for febrile children in European emergency departments

**DOI:** 10.1007/s00431-022-04417-8

**Published:** 2022-03-21

**Authors:** Lorenzo Zanetto, Josephine van de Maat, Daan Nieboer, Henriette Moll, Alain Gervaix, Liviana Da Dalt, Santiago Mintegi, Silvia Bressan, Rianne Oostenbrink

**Affiliations:** 1 Department of Women’s and Children’s Health, University of Padova, Padua, 35128 Italy; 2 Department of General Pediatrics, Erasmus Medical Center Sophia Children’s Hospital, Rotterdam, 3015 CN Netherlands; 3 Department of Pediatrics, Gynaecology and Obstetrics, University Hospital of Geneva, Geneva, 1205 Switzerland; 4 Pediatric Emergency Department, Biocruces Bizkaia Health Research Institute, Hospital Universitario Cruces, University of the Basque Country, UPV/EHU, Bilbao, Basque Country, Spain

**Keywords:** Children, Emergency department, Fever, Diagnosis, Blood testing, Variation

## Abstract

The study aimed to explore the use of diagnostics for febrile children presenting to European emergency departments (EDs), the determinants of inter-hospital variation, and the association between test use and hospitalization. We performed a secondary analysis of a cross-sectional observational study involving 28 paediatric EDs from 11 countries. A total of 4560 children < 16 years were included, with fever as reason for consultation. We excluded neonates and children with relevant comorbidities. Our primary outcome was the proportion of children receiving testing after primary evaluation, by country and by focus of infection. Variability between hospitals and effects of blood testing on patient disposition were explored by multilevel regression analyses, adjusting for patient characteristics (age group, triage level, appearance, fever duration, focus of infection) and hospital type (academic, teaching, other). The use of routine diagnostics varied widely, mostly in the use of blood tests, ranging from 3 to 75% overall across hospitals. Age < 3 months, high-acuity triage level, ill appearance, and suspicion of urinary tract infection displayed the strongest association with blood testing (odds ratios (OR) of 8.71 (95% CI 5.23–14.53), 19.46 (3.66–103.60), 3.13 (2.29–4.26), 10.84 (6.35–18.50), respectively). Blood testing remained highly variable across hospitals (median OR of the final model 2.36, 1.98–3.54). A positive association was observed between blood testing and hospitalization (OR 13.62, 9.00–20.61).

*Conclusion*: the use of diagnostics for febrile children was highly variable across European EDs, yet patient and hospital characteristics could only partly explain inter-hospital variability. Focus groups of participating sites should help define reasons for unexpected variation.

**Table:** 

**What is Known:** *• Although previous research has shown variation in the emergency department (ED) management of febrile children, there is limited information on the use of diagnostics in European EDs.* *• A deeper knowledge of variability and its determinants can steer optimization of care.*	
**What is New:** *• The use of diagnostics for febrile children was highly variable across European EDs, yet patient and hospital characteristics could only partly explain inter-hospital variability.* *• Data on between-centre comparison offer opportunities to further explore factors influencing unwarranted variation.*

**Supplementary information:**

The online version contains supplementary material available at 10.1007/s00431-022-04417-8.

## Introduction

One frequent challenge for emergency department (ED) physicians seeing the many children presenting with fever is to early identify those requiring antibiotic treatment either as inpatients or outpatients, while limiting invasive testing and avoiding antibiotics for those with a benign viral infection [1, 2]. Several research efforts have focused on diagnostic screening tools, including point-of-care tests, to help ED physicians face this challenge [3–5]. However, limited information is available on patterns of diagnostic test use for febrile children in the ED. The analysis of the current utilization of diagnostic tests for febrile children presenting to European EDs can be valuable to identify areas requiring interventions to optimize ED management at both patient and institution level [6, 7].

Previous studies have shown variation in the ED diagnostic and therapeutic management of febrile children in the USA, while European research has solely focused so far on antibiotic treatment [8–11]. The primary aims of this cross-sectional study are to describe the use of diagnostic tests in febrile children presenting to a broad set of European EDs and to identify factors associated with the use of diagnostic tests. As a secondary aim, we set out to evaluate whether test use in the ED influences patient disposition.

## Methods

### Study design and participants

We performed a planned secondary analysis of data from a multicentre cross-sectional observational study, including children aged 1 month to 16 years with fever as the reason for consultation [10], from 28 paediatric EDs in 11 countries, all members of the Research in European Pediatric Emergency Medicine (REPEM) network [12]. The characteristics of participating sites are reported in Supplemental Material 1.

The parent study aimed to investigate variability in antibiotics prescribed to febrile children in European EDs. Patients were excluded if they repeatedly visited the ED for the same problem within 7 days, if they had received antibiotics < 7 days before their visit, and if they had an antibiotic allergy. The present work only analyzed patients without relevant comorbidities. Comorbidities were classified as cardiovascular, respiratory, renal, haematological or immunological, neuromuscular, genetic defects, and malignancy and were defined as relevant by the responsible clinician or according to medical complexity [13].

We used the Strengthening the Reporting of Observational Studies in Epidemiology (STROBE) guidelines to report this study (Supplemental Material 6) [14].

### Procedures

The data collection took place from October 2014 to February 2016. Detailed procedures are described in the primary paper [10]. Participating EDs recorded medical information for all attending febrile children for one random day each month for 12 consecutive months. We collected data on blood screening tests (including white blood cell (WBC) count, C-reactive protein (CRP), procalcitonin (PCT)), blood culture, chest X-ray, urinalysis, and lumbar puncture. Hospital information was collected using a separate survey (Supplemental Material 2A/B). Data were extracted from patient records and reported in an electronic study case report form by each site investigator after the sampling day. Site investigators were informed of the general scope of the study as a registry of febrile children, but diagnostic testing and disposition were not known as outcomes of interest. The study was approved by the promoting centre’s ethics committee (Erasmus University: MEC-2014–419) and at each participating site.

### Outcome measures

Outcomes included hospital-level rates of diagnostic testing (blood tests, chest x-ray or urinalysis, by country and by focus of infection), and hospital admission rate on initial ED visit.

### Statistical analysis

We described the use of diagnostic tests, by hospital and presumed focus of infection.

We used a multilevel logistic regression model (using a random intercept for hospital) to calculate the influence of patient-level and hospital determinants on the use of blood screening tests. Results were reported as odds ratios (OR). For these analyses, children belonging to six groups with respect to the focus of infection were considered: children with (i) upper and (ii) lower respiratory tract infection, (iii) children with presumed enteric focus of infection, (iv) children with urinary tract infection (UTI), (v) children with fever of cutaneous origin, (vi) fever without source (FWS). Groups with fewer than 100 patients were excluded from these analyses. We excluded from the model analysis records with missing data on any of the tested determinants or on the outcome, as these were < 5% of the overall dataset. We used a flowchart to describe the patient selection process.

The null model included a random intercept for hospital only. Clinical variables were added sequentially. First, we included patient-level risk factors for serious bacterial infections, based on validated clinical prediction rules and guidelines from the UK National Institute for Health and Care Excellence [3, 15]. We did not include vital signs in the model due to high levels of missing data in our dataset. Their measurement has already been shown to be highly variable across EDs and are more often done depending on other patient-level determinants, such as age [16]. We however included other measures of general illness and severity such as triage level and ill appearance in the model. Second, the focus of infection was added to the analysis. Finally, hospital variables were selected depending on the following factors: (1) completeness of the data; (2) strength of the association between predictors and test use; (3) value of variables according to previous studies on the interpretation of observational data for ED quality of care [17].

We calculated the percentage of blood screening test use for each hospital on the basis of the null model (crude test use) and the final model (adjusted test use), illustrated by caterpillar plots. A number of 0 indicates the use of blood tests by the average hospital, a number above 0 indicates higher use than average, and a number below 0 means fewer blood tests being used than average. In order to quantify the between-hospital variation in rate of test use, we calculated the median odds ratio (MOR) for the null and the final model. The MOR represents the median increase in odds of receiving a blood screening test when moving from one hospital to another which has a higher use of blood screening tests. The MOR is directly comparable with the ORs of other patient-level variables included in the model [18].

We used a multilevel regression model to investigate the association between blood test use and hospitalization, adjusting for patient and hospital characteristics. Analyses were conducted in SPSS Statistic (version 24) and R (version 4.0.2) [19, 20].

## Results

We included 4560 patients in the descriptive analysis [10, 16]. Median age was 2.4 years (IQR 1.1–4.7) and 2451 (54%) were male. Age groups distributed similarly across countries, with the majority of children being between 1 and 5 years of age. Age distribution was highly variable when reported by foci of infection (Supplemental Material 3). Detailed data on patient characteristics are reported in Table 1.

**Table 1:** Baseline characteristics of the enrolled population

Patients (*n* = 4560)	
	**Proportion of patients (*n* (%))**
**General characteristics**	
Male sex	2451 (54%)
Mean age (years)	2.4 (1.1–4.7)
Age groups	
1–3 months	160 (4%)
3 months–1 year	867 (19%)
1–5 years	2479 (54%)
> 5 years	1054 (23%)
Method of referral	
General practitioner	395 (9%)
Self	3966 (87%)
Other	163 (4%)
Triage level	
Immediate or very urgent	197 (4%)
Urgent	1042 (23%)
Standard	1866 (41%)
Non-urgent	745 (16%)
Ill appearance	431 (10%)
**Diagnostic assessment**	
WBC count and/or CRP testing	810 (18%)
PCT testing^a^	141 (3%)
Blood culture	224 (5%)
Chest X-ray	431 (8%)
Urinalysis	841 (18%)
Lumbar puncture	34 (1%)
**Focus of infection**	
Upper respiratory tract	2821 (62%)
Lower respiratory tract	486 (11%)
Enteric	531 (12%)
Urinary tract	125 (3%)
Cutaneous	116 (3%)
Fever without source	284 (6%)
Viral childhood illness	30 (1%)
Sepsis/meningitis	15 (< 1%)
Bone/joint	12 (< 1%)
Inflammatory disease	7 (< 1%)
Other	34 (1%)
**Working diagnosis**	
Definite viral	494 (11%)
Probable viral	2405 (53%)
Definite bacterial	198 (4%)
Probable bacterial	1198 (26%)
Uncertain	235 (5%)
Other	14 (< 1%)
**Treatment/disposition**	
Antibiotic prescription	1454 (32%)
Disposition	
Discharged	4035 (88%)
Observation unit < 24 h	187 (4%)
Admitted to ward	321 (7%)
Admitted to intensive care unit	11 (< 1%)

Missing data: gender, age, method of referral, working diagnosis, treatment/disposition ≤ 1%, appearance < 1.5%, focus of infection 2%, triage level 16%. Triage level was not available for 96% of the Turkey’s patients (683/708)

Abbreviations: *WBC*, white blood cell count; *CRP*, C-reactive protein; *PCT*, procalcitonin

^a^Only as additional testing to white blood cell count and/or CRP testing

**Fig. 1:**
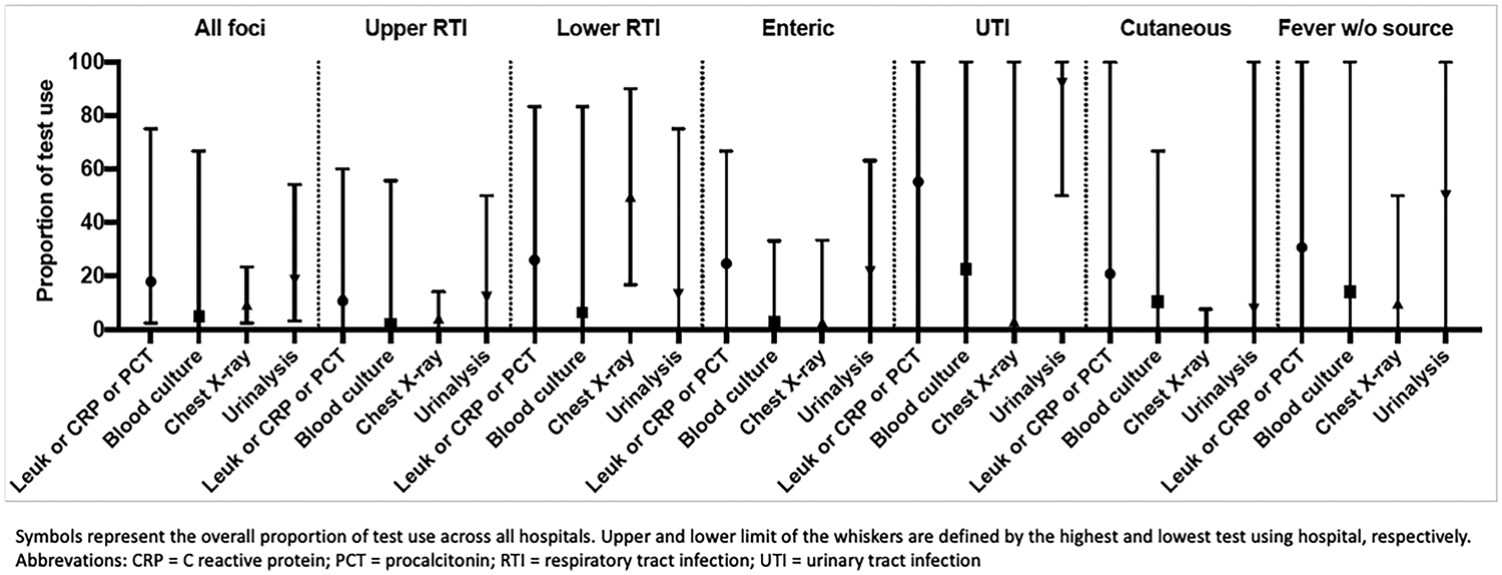
Proportions of test use by focus of infection

Urinalysis and blood screening tests were both performed in 18% of cases (841/4560 and 810/4560, respectively). PCT was available in 15 of 28 centres and was performed in 5.5% of children (141/2542), always in conjunction with other blood screening tests. Use of diagnostic tests varied widely across hospitals and by focus of infection (Fig. 1). The percentage of blood screening testing was the highest in patients with UTI (69/125 [55%]) and FWS (87/284 [31%]). Most of blood cultures and urinalyses were performed on patients with FWS (14% and 50%, respectively) and presumed UTI (22% and 92%, respectively). Four hundred thirty-one (9%) received a chest x-ray. The use of chest x-ray was the highest (241/487 [50%]) for patients with lower respiratory tract infections. Overall 34/4560 (0.7%) children underwent lumbar puncture.

A total of 1454 (32%) children were prescribed antibiotics and 332 (7%) were hospitalized, while 3098 (68%) were discharged home without diagnostic tests being performed.

**Fig. 2:**
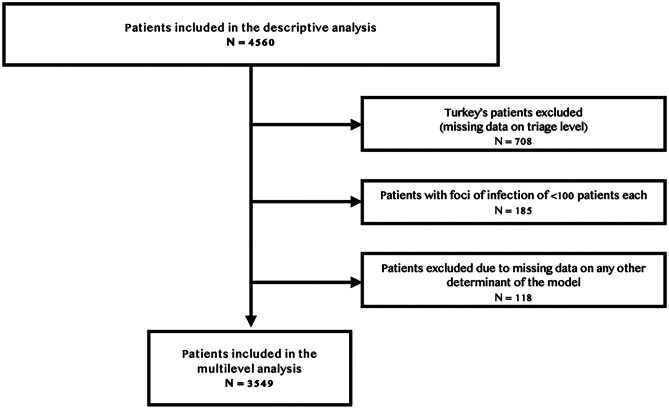
Flowchart of patient selection

The multilevel analysis was performed on 3549 records from 27 of 28 hospitals. The patient selection process is detailed in Fig. 2. Patients from Turkey (708/4560 [15.5%]) were excluded, because of missing data on triage level. Results of the final model are described in Table 2. Age < 3 months, highest priority triage level, ill appearance, and UTI as the focus of fever displayed the strongest association with blood testing (ORs of 8.71 (95% CI 5.23–14.53), 19.46 (95% CI 3.66–103.60), 3.13 (95% CI 2.29–4.26), 10.84 (95% CI 6.35–18.50), respectively). The inclusion of hospital-associated factors yielded the best model fit. However, these factors were not significantly associated with blood tests use. Results were not altered by the exclusion of patients from Turkey, as shown by the exploratory models, available as Supplemental material 4.

**Table 2:** Determinants of blood testing in febrile children

Level	Determinants	OR (95% CI)
**Patient characteristics**	Intercept	0.01 (0.001–0.09)
	Age group (> 5 years: reference)	
	< 3 months	8.71 (5.23–14.53)
	3 months–1 year	1.02 (0.74–1.39)
	1–5 years	0.80 (0.61–1.04)
	Triage level (non-urgent: reference)	
	Immediate	19.46 (3.66–103.60)
	Very urgent	7.64 (4.34–13.49)
	Urgent	4.58 (3.11–6.74)
	Standard	1.53 (1.06–2.22)
	Fever duration in days^a^	1.41 (1.31–1.51)
	Ill appearance (well-appearance: reference)	3.13 (2.29–4.26)
	Focus of infection (upper respiratory tract: reference)	
	Lower respiratory tract	1.37 (1.01–1.85)
	Enteric	2.33 (1.76–3.09)
	Urinary tract	10.84 (6.35–18.50)
	Cutaneous	1.89 (1.09–3.28)
	Fever without source	3.03 (2.09–4.39)
**Hospital characteristics**	Hospital type (non-teaching: reference)	
	Academic	3.70 (0.41–33.31)
	Teaching	2.32 (0.25–21.54)

Abbreviations: *OR*, odds ratio; *CI*, confidence interval

^a^An upper limit of five days duration was set for modelling purposes

**Fig. 3:**
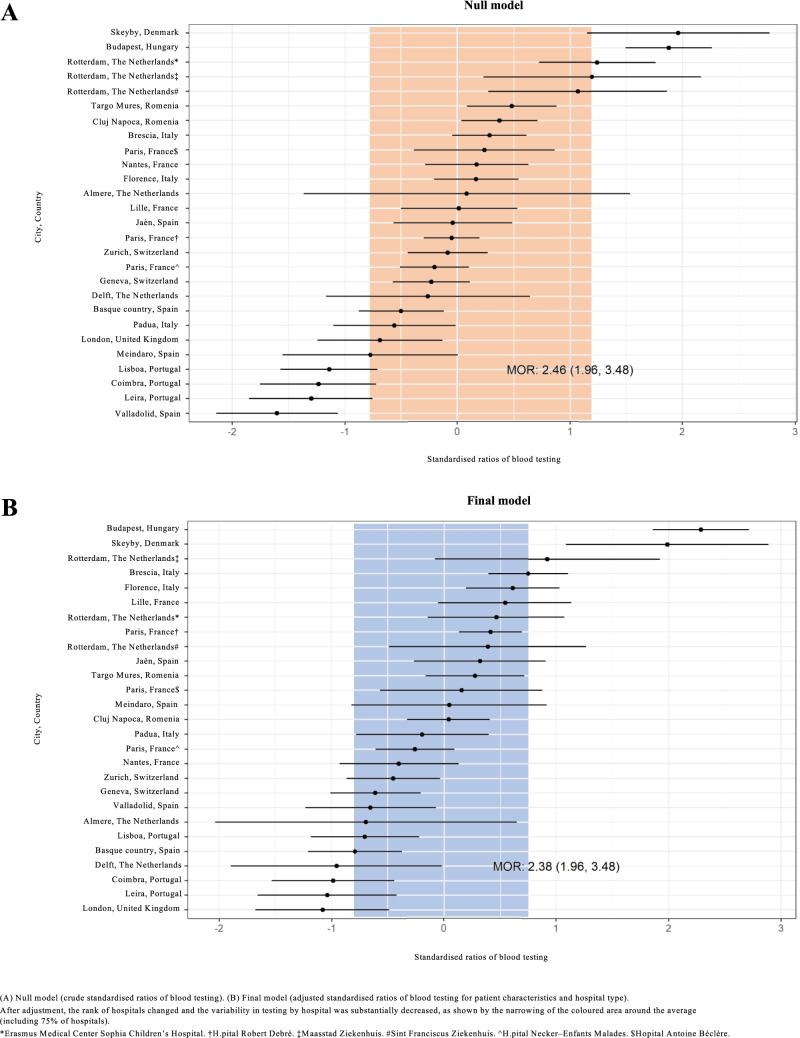
Standardized ratios of blood testing per hospital

Figure 3A presents the crude ratios of blood testing for each included hospital based on the null model. Figure 3B shows adjusted testing ratios based on the final model. After adjustment, the rank of hospitals changed and the variability in testing by hospital was substantially decreased, as shown by the narrowing of the coloured area around the average (including 75% of hospitals). However, variability in the use of blood screening tests remained, as shown by the caterpillar plot and the MOR value (Fig. 3B). The MOR based on the final model was 2.36 (CI 95% 1.96–3.48). That is, if a person moves from one hospital to another hospital with a higher probability of performing blood screening tests, his/her probability of receiving blood tests will (in median) increase 2.36 times. The residual heterogeneity between hospitals (MOR = 2.36) was of greater relevance than was the impact of other determinants, e.g. of fever duration (OR = 1.41) or of having a lower respiratory tract infection (OR = 1.37).

**Fig. 4:**
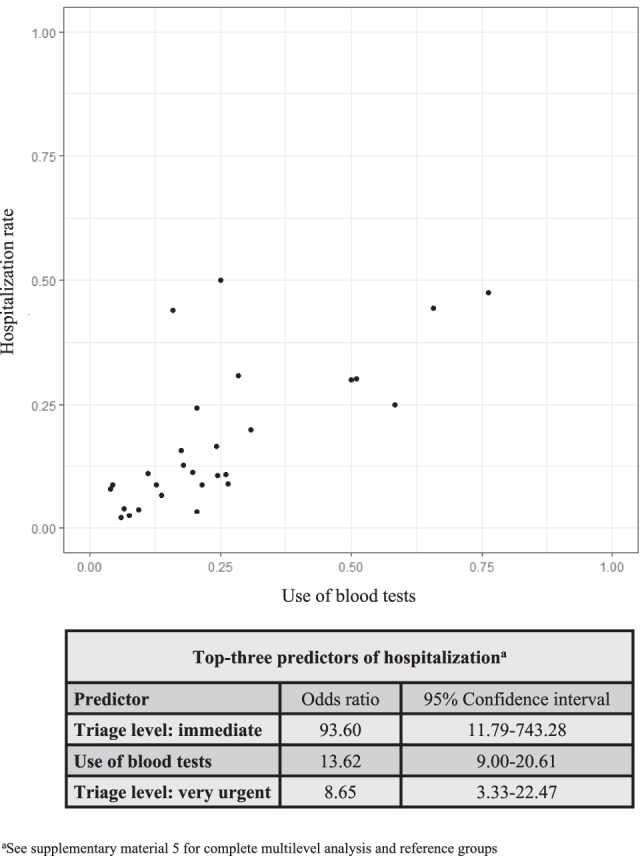
Correlation between test use and hospitalization

Figure 4 shows the correlation between raw percentages of blood testing and hospitalization. To better interpret the correlation, we performed a multilevel analysis, adjusting for patient characteristics and hospital type. The analysis showed that the use of blood tests remained highly associated with hospitalization even after adjustment for relevant factors (OR 13.62; 95% CI 9.00–20.61; Fig. 4; the complete analysis is included as Supplemental Material 5).

## Discussion

Our study prospectively investigated the use of diagnostic tests in febrile children attending 28 European EDs in 11 countries, with consideration of variability and its determinants, overall and by focus of infection. This had the specific aim of understanding pathways and reasons for variation in clinical use of diagnostic tests, in order to identify areas for improvement in the management of febrile children. Younger age, UTI as focus of fever, and high-urgency triage level showed the strongest association with blood testing. However, the factors included in the multilevel analysis could only explain part of the variability observed across hospitals. The observed diversity in testing rates (even when adjusted for patient characteristics, focus of infection, and hospital characteristics) suggests room for further investigation and optimization of ED management. In addition, the analyses showed a positive association between the use of diagnostic tests and hospitalization, even after adjusting for patient and hospital characteristics.

There have been previous reports on the frequency of diagnostic testing for febrile children in paediatric EDs outside Europe. The work by Khine et al. from the USA compared a single paediatric ED and a general ED, with respect to management of well-appearing febrile children [21], finding a lower use of complete blood count (in 8 of 224 included children, 4%) than in our study (14% of the subgroup of well-appearing children). The use of blood cultures and chest X-rays was similar to our report. The North-American study also found a high use of rapid viral testing (45% of 224 children), likely related with the concurrent 2009 H1N1 outbreak.

The study by Goldman et al. involving 6 paediatric EDs across Canada focused on patients < 3 months of age, reporting large homogeneity in blood and urine testing (from 83 to 95% and from 78 to 95%, respectively), with high variation in lumbar puncture use (from 25 to 62% of children) [11]. The use of chest x-rays was even more variable (12 to 62% across centres), with higher use than in our cohort, as expected due to the different populations.

The most recent study by Aronson et al. focused on the same age group of young febrile infants showing the expected decrease of lumbar puncture use with older age (from 72% in patients < 1 month to 13.1% in infants > 2 months). The use of blood tests remained high (> 75% of patients) throughout the first 2 months of life, while the choice of sole urine testing significantly increased for patients in the third month of life [9].

A recent European multicentre study by Hagedoorn et al. further analyzed variation in antibiotic prescription rates in febrile children presenting to 12 EDs across 8 countries [6]. Forty-five percent of 35,650 included patients were tested for CRP, 25% received urinalysis, and 14% chest X-ray, with the largest variation for CRP use (7–92% of patients across centres). In addition to these studies, our study explored the reasons behind variability in diagnostic tests use in a wide population of febrile children > 1 month of age.

It was not the aim of this study to evaluate the correct use of diagnostic tests according to the existing guidelines on febrile children’s management in the ED. We however knew that most of the participating centres used NICE guidelines or a national adaptation [15, 22, 23]. We expected and found an overall higher use of blood tests for patients with UTI and FWS, and a lower use for patients with upper respiratory tract infections. Nevertheless, substantial variability could be noticed across hospitals for each of these foci (Fig. 1). The same was observed for the use of urinalysis, especially when respiratory tract infections were suspected for patients with FWS. The following analyses showed that wide variation in blood testing remained even when adjusted for characteristics of illness severity. Overall, these findings emphasize the need for a more standardized and rationalized testing across European EDs. This should be reached through cooperation of participating sites’ leads in focus groups, aiming to identify the system factors contributing to diverse patient management, especially in those hospitals that placed farthest from the average (Fig. 3). Specific considerations should also be made to identify reasons behind the high use of urinalysis in patients with presumed respiratory tract infections (up to 60%, Fig. 1) and high use of blood tests for patients with presumed UTI, in order to reduce unnecessary testing for well-appearing children not warranting hospitalization [22, 24]. Fewer considerations could be made on the use of blood tests for presumed enteric focus, as some of these patients may receive tests at the time of catheter placement for rehydration purposes. Finally, across foci of infection, the lowest variability could be observed for the use of chest X-rays and blood cultures. Only 10% (28/284) of patients with FWS received a chest X-ray, even if 77/284 children < 5 years satisfied intermediate/high risk signs or symptoms for whom the NICE guidelines recommend this investigation [15].

There have been previous reports of ED testing to influence the decision to hospitalize [8]. The reported odd for the association between use of blood tests and hospitalization was comparable to our findings (OR 10.4 (95% CI 10–10.8) for complete blood count and OR 7.3 (95% CI 6.9–7.7) for inflammatory markers). We similarly observed that hospitals with higher use of diagnostic tests showed higher hospitalization rates, even when adjusted for characteristics of illness severity and hospital-level characteristics in a statistical model that accounted for clustering.

Most of the study strengths and limitations are shared in the primary paper. Hospitals were invited through the REPEM network, which ensured broad participation and high-quality data. The involvement of 11 countries enabled comparisons across a large part of Europe. Methods for data collection were designed in order to minimize biases, as detailed in the primary paper [10]. Although we acknowledge that some variability may occur in patient assessment at both triage and medical visit [25], several other determinants were included in the model and senior clinician supervision ensured the highest possible quality in the assessment of patient appearance. There are some limitations to our approach. First, we could not investigate the influence of rapid viral tests availability on the use of other testing in the ED, as data on viral testing was not gathered by the primary study. Second, we could not explore variability with multilevel analyses for children with sepsis, meningitis, bone and joint infections, inflammatory diseases, and viral childhood illnesses due to low numbers of children in these categories. However, we observed less variation in the use of blood screening tests in these groups. Third, our multilevel analysis could not include data on vital signs, due to missing values. However, other measures of general illness such as triage level and ill appearance were included in our models. Fourth, the number of included hospitals per country does not match the country’s population size and four of 11 countries participated with only a single hospital. Hence, we were not able to take clustering at country level into account. Fifth, some hospitals had small sample sizes (five hospitals included < 50 patients), thereby limiting the power to show large differences between hospitals. An additional limitation is that no follow-up data are available on included patients; thus, no conclusions on the effect of variation on the long-term outcome of febrile children can be inferred. Finally, the development of our models largely employed UK NICE tools of risk quantification, as the early identification of serious bacterial infection has been a primary challenge for ED physicians and the most relevant at the time of data collection. However, viral infections or non-infectious causes of fever can sometimes have serious consequences. When these conditions are suspected, blood tests are performed to look for possible organ disfunction, along with inflammatory markers. Thus, the need for investigation may rise from other factors beyond those related to the need to identify or exclude serious bacterial infections.

## Conclusion

The use of diagnostic tests for febrile children is highly variable across European EDs, yet patient and hospital characteristics can only partly explain inter-hospital variability. Future studies shall explore reasons behind unexplained variability of diagnostic management. Focus groups of participating sites should help define reasons for unexpected variation and steer European EDs towards a more rationalized test use. Next, this variability and its determinants should be addressed in studies evaluating the impact of diagnostic tests use on health outcomes of febrile children. This future research may contribute to reduce ED and hospital length of stay, decrease costs, and prevent unnecessary hospitalizations.

## Supplementary information

Below is the link to the electronic supplementary material.

Supplementary file1 (DOCX 770 kb)

## Data Availability

Data will not be accessible from a data repository. Data are available on request to corresponding author, with a proposal including research question.

## List of abbreviations

CI: confidence interval
CRP: C reactive protein
ED: emergency department
FWS: fever without source
PCT: procalcitonin
REPEM: Research in European Pediatric Emergency Medicine
UTI: urinary tract infection
WBC: white blood cell

## Declarations

### Disclaimer

All authors affirm that this manuscript is an honest, accurate, and transparent account of the study being reported; that no important aspects of the study have been omitted; and that any discrepancies from the study as planned (and, if relevant, registered) have been explained.
