# Supplementary material for: Diagnostic variation for febrile children in European emergency departments
Source: Eur J Pediatr. 2022 Mar 21;181(6):2481–90. doi: 10.1007/s00431-022-04417-8 (PMC9110537; doi:10.1007/s00431-022-04417-8)
Supplement: Supplementary file 1 — Supplementary file1 (DOCX 770 kb) [file 431_2022_4417_MOESM1_ESM.docx]

**Title:** Diagnostic variation for febrile children in European emergency departments

**Authors:** Lorenzo Zanetto^a^, Josephine van de Maat^b^, Daan Nieboer^b^, Henriette Moll^b^, Alain Gervaix^c^, Liviana Da Dalt^a^, Santiago Mintegi^d^, Silvia Bressan^a^, Rianne Oostenbrink^b^

**Affiliations:**

^a^Department of Women’s and Children’s Health, University of Padova, 35100 Padova, Italy

^b^Department of General Pediatrics, Erasmus Medical Center Sophia Children’s Hospital, 3015 CN Rotterdam, Netherlands

^c^Department of Pediatrics, Gynaecology and Obstetrics, University Hospital of Geneva, 1205 Geneva, Switzerland

^d^Pediatric Emergency Department, Biocruces Bizkaia Health Research Institute, Hospital Universitario Cruces. University of the Basque Country, UPV/EHU. Bilbao, Basque Country, Spain

**Corresponding Author e-mail address:** [silviabress@gmail.com](mailto:silviabress@gmail.com)

**ABSTRACT**

The study aims to explore the use of diagnostics for febrile children presenting to European emergency departments (EDs), the determinants of inter-hospital variation, and the association between test use and hospitalisation.

We performed a secondary analysis of a cross-sectional observational study involving 28 paediatric EDs from 11 countries. 4560 children <16 years were included, with fever as reason for consultation. We excluded neonates and children with relevant comorbidities. Our primary outcome was the proportion of children receiving testing after primary evaluation, by country and by focus of infection. Variability between hospitals and effects of blood testing on patient disposition were explored by multilevel regression analyses, adjusting for patient characteristics (age group, triage level, appearance, fever duration, focus of infection) and hospital type (academic, teaching or other).

The use of routine diagnostics varied widely, mostly in the use of blood tests, ranging from 3% to 75% overall across hospitals. Age <3 months, high-acuity triage level, suspicion of urinary tract infection and fever without source displayed the strongest association with blood testing [Odds ratios (OR) of 8.71 (95% CI 5.23 to 14.53), 19.46 (3.66 to 103.60), 10.84 (6.35 to 18.50), 3.03 (2.09 to 4.39) respectively]. Blood testing remained highly variable across hospitals (median OR of the final model 2.36, 1.98 to 3.54). A positive association was observed between blood testing and hospitalisation (OR 13.62, 9.00 to 20.61).

*Conclusion*: the use of diagnostics for febrile children was highly variable across European EDs, yet patient and hospital characteristics could only partly explain inter-hospital variability.

**Keywords**

Children

Emergency department

Fever

Diagnosis

Blood testing

Variation

___________________________________________________________________________________________________

**Supplementary material index**

S 1: Baseline characteristics of the hospitals

S 2: Data collection form

S 3: Distribution of age groups by country and by focus of infection

S 4: Multilevel models for test performance including Turkey

S 5: Predictors of hospitalisation

S 6: STROBE checklist

___________________________________________________________________________________________________

Supplementary material 1. Baseline characteristics of the hospitals

| **Hospital** | **Country (code)** | **n** | **Type** | **Setting** | **Paediatric healthcare system** | **Responsible specialist** | **Mode of supervision** | **Hib coverage** | **PCV coverage** |
| --- | --- | --- | --- | --- | --- | --- | --- | --- | --- |
| Aarhus Universitetshospital, Skejby | Denmark (DK) | 32 | Academic | Mixed | GP system | Paediatrician | Indirect/combination | >90 | >90 |
| Hopital Antoine Béclère, Paris | France (FR) | 58 | Academic | Inner city | Combined system | NA | NA | >90 | >90 |
| Hôpital Mère-Enfant, Nantes |  | 153 | Academic | Inner city |  | Paediatrician | Direct | >90 | >90 |
| Hôpital Necker-Enfants malades, Paris |  | 333 | Academic | Inner city |  | PEM specialist | Direct | >90 | >90 |
| Hopital Robert Debre, Paris |  | 431 | Academic | Inner city |  | Paediatrician | Direct | >90 | >90 |
| Roger Salengro Hospital, Lille |  | 103 | Teaching | Inner city |  | PEM specialist | Direct | >90 | >90 |
| Heim Pal Children's Hospital, Budapest | Hungary (HU) | 135 | Teaching | Mixed | Combined system | Paediatrician | Direct | >90 | >90 |
| Meyer University Children's Hospital, Florence | Italy (IT) | 175 | Academic | Inner city | Combined system | Paediatrician | Direct | >90 | 87-89 |
| Ospedale dei Bambini, Azienda Ospedaliera Spedali Civili, Brescia |  | 195 | Academic | Mixed |  | Paediatrician | Direct | >90 | 87-89 |
| University Hospital, Padova |  | 116 | Academic | Inner city |  | Paediatrician | Indirect/combination | >90 | 87-89 |
| ErasmusMC – Sophia, Rotterdam | The Netherlands (NL) | 99 | Academic | Inner city | GP system | Paediatrician | Indirect/combination | >90 | >90 |
| Flevoziekenhuis, Almere |  | 26 | Teaching | Mixed |  | Paediatrician | Indirect/combination | >90 | >90 |
| Maasstad Ziekenhuis, Rotterdam |  | 34 | Teaching | Inner city |  | Paediatrician | Indirect/combination | >90 | >90 |
| Reinier de Graaf, Delft |  | 40 | Teaching | Mixed |  | Paediatrician | Indirect/combination | >90 | >90 |
| Sint Franciscus Ziekenhuis, Rotterdam |  | 26 | Teaching | Inner city |  | Paediatrician | Indirect/combination | >90 | >90 |
| Centro Hospitalar de Leiria, Leiria | Portugal (PT) | 215 | Teaching | Mixed | Combined system | Paediatrician | Direct | >90 | NA |
| Lisbon Medical Academic Center (Hospital de Santa Maria), Lisboa |  | 353 | Academic | Inner city |  | Paediatrician | Direct | >90 | NA |
| Hospital Pediátrico, Centro Hospitalar e Universitário de Coimbra |  | 275 | Teaching | Inner city |  | Paediatrician | Direct | >90 | NA |
| Emergency Children's Hospital, Cluj Napoca | Romania (RO) | 179 | Teaching | Regional | GP system | Paediatrician or PEM | Direct | >90 | NA |
| Tirgu Mures Emergency Clinical County Hospital, Tirgu Mures |  | 122 | Academic | Inner city |  | Paediatrician | Direct | >90 | NA |
| Cruces University Hospital Bilbao, Basque country | Spain (ES) | 277 | Academic | Inner city | Paediatric system | PEM specialist | Indirect/combination | >90 | NA |
| Hospital de Mendaro, Mendaro (Guipúzcua) |  | 71 | Non-teaching | Regional |  | NA | NA | >90 | NA |
| Hospital Universitario Rio Hortega, Valladolid |  | 266 | Teaching | Mixed |  | PEM specialist | Indirect/combination | >90 | NA |
| San Agustín University Hospital, Linares, Jaén |  | 106 | Teaching | Mixed |  | Paediatrician | Indirect/combination | >90 | NA |
| University Hospital, Geneva | Switzerland (CH) | 265 | Academic | Inner city | Combined system | PEM specialist | Direct | >90 | 80-81 |
| Children’s Hospital of Zurich, Zurich |  | 205 | Academic | Inner city |  | Paediatrician or PEM | Direct | >90 | 80-81 |
| Cukurova University Medical Faculty Balcali Hospital, Adana | Turkey (TK) | 740 | Academic | Mixed | Combined system | PEM specialist | Direct | >90 | >90 |
| St Mary's Hospital, London | United Kingdom (UK) | 147 | Academic | Inner city | GP system | PEM specialist | Indirect/combination | >90 | >90 |

*GP = general practitioner; PEM = paediatric emergency medicine; NA = not available*

Supplemental material 2: Data collection form

1. Patient data

1. Hospital data

Supplemental material 3: Distribution of age groups by country and by focus of infection

Supplemental material 4: Multilevel models for blood testing including Turkey

Supplemental material 5: Predictors of hospitalisation

| **Predictor** | **OR** | **95% CI** |
| --- | --- | --- |
| Age <3 months | 2.41 | 1.17-4.98 |
| Age 3 months-1 year | 1.84 | 1.05-3.22 |
| Age 1-5 years | 1.04 | 0.64-1.70 |
| Age >5 years | reference | |
| Triage level: immediate | 93.60 | 11.79-743.28 |
| Triage level: very urgent | 8.65 | 3.33-22.47 |
| Triage level: Urgent | 3.21 | 1.43-7.22 |
| Triage level: Standard | 0.94 | 0.39-2.23 |
| Triage level: Non-urgent | Reference | |
| Fever duration | 0.89 | 0.78-1.00 |
| Ill-appearance | 2.09 | 1.39-3.15 |
| Well-appearance | reference | |
| Use of blood tests | 13.62 | 9.00-20.61 |
| No testing | reference | |
| Focus: Fever without source | 0.64 | 0.27-1.48 |
| Focus: Lower respiratory tract infection | 5.52 | 3.40-8.97 |
| Focus: Enteric | 3.91 | 2.33-6.54 |
| Focus: Urinary tract infection | 1.30 | 0.57-2.93 |
| Focus: Cutaneous | 3.11 | 1.27-7.64 |
| Focus: Upper respiratory tract infection | reference | |
| Working diagnosis: probable viral | 0.61 | 0.32-1.18 |
| Working diagnosis: uncertain | 2.54 | 1.03-6.27 |
| Working diagnosis: probable bacterial | 1.35 | 0.67-2.71 |
| Working diagnosis: definite bacterial | 3.31 | 1.44-7.61 |
| Working diagnosis: definite viral | reference | |
| Hospital type: academic | 2.31 | 0.16-32.85 |
| Hospital type: teaching | 5.89 | 0.41-85.54 |
| Hospital type: non-teaching | reference | |
| Abbreviations: OR: Odds ratio; CI = Confidence interval | |  |

Supplemental material 6: STROBE Statement—Checklist of items that should be included in reports of cross-sectional studies

|  | Item No | Recommendation | Page No |
| --- | --- | --- | --- |
| Title and abstract | 1 | (*a*) Indicate the study’s design with a commonly used term in the title or the abstract | 1 |
|  |  | (*b*) Provide in the abstract an informative and balanced summary of what was done and what was found | 1 |
| Introduction | | | |
| Background/rationale | 2 | Explain the scientific background and rationale for the investigation being reported | 2 |
| Objectives | 3 | State specific objectives, including any prespecified hypotheses | 2 |
| Methods | | | |
| Study design | 4 | Present key elements of study design early in the paper | 2 |
| Setting | 5 | Describe the setting, locations, and relevant dates, including periods of recruitment, exposure, follow-up, and data collection | 3 |
| Participants | 6 | (*a*) Give the eligibility criteria, and the sources and methods of selection of participants | 3 |
| Variables | 7 | Clearly define all outcomes, exposures, predictors, potential confounders, and effect modifiers. Give diagnostic criteria, if applicable | 3 |
| Data sources/ measurement | 8* | For each variable of interest, give sources of data and details of methods of assessment (measurement). Describe comparability of assessment methods if there is more than one group | 3-4 |
| Bias | 9 | Describe any efforts to address potential sources of bias | 3, parent study |
| Study size | 10 | Explain how the study size was arrived at | n.a. |
| Quantitative variables | 11 | Explain how quantitative variables were handled in the analyses. If applicable, describe which groupings were chosen and why | 4 |
| Statistical methods | 12 | (*a*) Describe all statistical methods, including those used to control for confounding | 3-4 |
|  |  | (*b*) Describe any methods used to examine subgroups and interactions | 4 |
|  |  | (*c*) Explain how missing data were addressed | 3-4 |
|  |  | (*d*) If applicable, describe analytical methods taking account of sampling strategy | n.a. |
|  |  | (*e*) Describe any sensitivity analyses | n.a. |
| Results | | | |
| Participants | 13* | (a) Report numbers of individuals at each stage of study—eg numbers potentially eligible, examined for eligibility, confirmed eligible, included in the study, completing follow-up, and analysed | Page 4, Table 1, flowchart (fig 2) |
|  |  | (b) Give reasons for non-participation at each stage | n.a. |
|  |  | (c) Consider use of a flow diagram | Fig 2 |
| Descriptive data | 14* | (a) Give characteristics of study participants (eg demographic, clinical, social) and information on exposures and potential confounders | Table 1, table 1 |
|  |  | (b) Indicate number of participants with missing data for each variable of interest | Table 1, flowchart suppl mat. |
| Outcome data | 15* | Report numbers of outcome events or summary measures |  |
| Main results | 16 | (*a*) Give unadjusted estimates and, if applicable, confounder-adjusted estimates and their precision (eg, 95% confidence interval). Make clear which confounders were adjusted for and why they were included | 4-5; table 2; suppl mat. |
|  |  | (*b*) Report category boundaries when continuous variables were categorized | Suppl mat |
|  |  | (*c*) If relevant, consider translating estimates of relative risk into absolute risk for a meaningful time period | n.a. |
| Other analyses | 17 | Report other analyses done—eg analyses of subgroups and interactions, and sensitivity analyses | 5 |
| Discussion | | | |
| Key results | 18 | Summarise key results with reference to study objectives | 5-6 |
| Limitations | 19 | Discuss limitations of the study, taking into account sources of potential bias or imprecision. Discuss both direction and magnitude of any potential bias | 7-8 |
| Interpretation | 20 | Give a cautious overall interpretation of results considering objectives, limitations, multiplicity of analyses, results from similar studies, and other relevant evidence | 6-7 |
| Generalisability | 21 | Discuss the generalisability (external validity) of the study results | 7 |
| Other information | | | |
| Funding | 22 | Give the source of funding and the role of the funders for the present study and, if applicable, for the original study on which the present article is based | 9 |

*Give information separately for exposed and unexposed groups.

**Note:** An Explanation and Elaboration article discusses each checklist item and gives methodological background and published examples of transparent reporting. The STROBE checklist is best used in conjunction with this article (freely available on the Web sites of PLoS Medicine at http://www.plosmedicine.org/, Annals of Internal Medicine at http://www.annals.org/, and Epidemiology at http://www.epidem.com/). Information on the STROBE Initiative is available at www.strobe-statement.org.
